# Efficacy and safety of Cook staged Extubation Set in patients with difficult airway: a systematic review and meta-analysis

**DOI:** 10.1186/s12871-023-02191-0

**Published:** 2023-07-07

**Authors:** Cheng Lu, Jian Li, Shibing Zhao, Yajun Zhang

**Affiliations:** Department of Anesthesiology, China-Japan Friendship Hospital, Beijing, 100029 China

**Keywords:** Difficult airway, Cook staged Extubation Set, Meta-analysis, Systematic review

## Abstract

**Background:**

Cook Stage extubation is a tool developed by Cook Medical for patients with difficult airways. Multiple clinical studies demonstrated the effectiveness and safety of Cook Stage extubation Set (CSES). Currently, no systematic review evidence has been published in this field. Therefore, this study aimed to review the clinical success rate, safety, and tolerability of CSES in patients with difficult airways.

**Method:**

The inclusion criteria were based on the population, intervention, comparator, outcomes, and study designs. An electronic search was conducted, and the following databases were used: PubMed, EMBASE, Cochrane Library, and Web of Science. Search keywords included difficult airway and CSES. The primary outcome was the CSES clinical success rate.The Joanna Briggs Institute Critical Appraisal tools for Case Series were used to assess the risk of bias in the included studies. R studio, version 4.2.2. was used to perform the statistical analysis. The Cochrane Q and *I*^2^ statistics were used to test the heterogeneity among all studies. Details of the included case reports were summarized in the systematic review part.

**Results:**

Five studies were eligible for meta-analysis, and 7 case reports were included for systematic review. The pooled overall CSES clinical success rate was 93% (95% CI: 85%, 97%). The CSES intolerable and complication incidence rates were 9% (95% CI: 5%, 18%) and 5% (95% CI: 2%, 12%), respectively. CSES clinical success rate was influenced by the study center and study design. The success rate of CSES was higher in multicenter and prospective design studies. Seven case reports have documented the successful operation of CSES intubation in obese, tall, oncologist, and pediatric patients.

**Discussion:**

This meta-analysis suggested that CSES have achieved a high clinical success rate in adult and pediatric patients with different physical conditions and types of surgery. The results of all original studies and meta-analysis confirmed a remarkably high tolerance rate and low overall complication rate. However, regardless of the tools chosen, a personalized, safe intubation strategy and a highly qualified anesthesiologist should be considered as the fundamental guarantee of a high clinical success rate. Future studies should also focus on the success rate of reintubation using CSES in patients with airway difficulties.

**Supplementary Information:**

The online version contains supplementary material available at 10.1186/s12871-023-02191-0.

## Background

Difficult airway generally refers to a clinical situation in which healthcare providers skilled in airway management experience difficulty using one or more standard airway management methods [1]. There is no standardized definition of the difficult airway, and available expert guidelines vary between countries. The 2022 American Society of Anesthesiologists Guidelines defines a difficult airway as “a conventionally trained anesthesiologist experiences difficulty with facemask ventilation of the upper airway, difficulty with tracheal intubation, or both [2].” Some definition is broader: “an experienced provider anticipates or encounters difficulty with any or all of face mask ventilation, direct or indirect (e.g., video) laryngoscopy, tracheal intubation, SGD (supraglottic device) use, or surgical airway [3].” For difficult airway patients, tracheal extubation is a high-risk stage of anesthesia. Most problems that occur during extubation and removal are minor, but a small and significant number can result in injury or death [4]. In response to this issue, the 1998 and 2005 Italian Guidelines [5], 2013 ASA Practice Guidelines [6], Canadian Guidelines [7], and the Difficult Airway Society extubation Guidelines [4] all discussed using airway exchange catheters (AECs) as part of a “safe extubation protocol.”

In addition to the AEC-based safe extubation strategy, Cook Medical has developed a new tool: Cook Stage extubation Set (CSES; Cook Intensive Care Unit; Bloomington, IN, USA). As an advanced version of AEC technology, CSES also allows for a phased extubation method. The CSES provides a guide wire that remains in place after extubation to maintain airway access and a reintubation catheter that can provide a catheter through the guide wire to track the tracheal tube or deliver oxygen through a central channel. By allocating corresponding marks on the endotracheal tube, depth marks are provided to facilitate correct and safe positioning. The staged principle of CSES is that the guide wire can be left in place to minimize side effects and improve patient compliance and that a dedicated hollow AEC (with absolutely little or no clearance from the endotracheal tube) can be used when reintubation is required.

The first large sample observational study of CSES was published in 2013, demonstrating the technique’s reliability and ease of use in adult patients with airway difficulties, with a reintubation failure rate of 13.8%. However, the authors also suggest that the risk of airway injury and complications (pneumothorax) associated with CSES is significant [8]. Currently, no meta-analysis has been published in this area. Therefore this study aimed to review the clinical success rate, safety, and tolerability of CSES in patients with a difficult airways.

## Methods

### Research design

The present meta-analysis was performed according to the Preferred Reporting Items for Systematic Reviews and Meta-analyses (PRISMA) statement [9].

### Search strategy and data sources

The data sources include these electronic databases: PubMed, EMBASE, Cochrane Library, and Web of Science (up to 14 Feb 2023). The following keywords were used: *((Airway exchange catheters) or (Cook Stage extubation Set) or (Tracheal exchange)) AND (Intubation intratracheal) AND (Difficult airway)*. Supplementary Table 1 documented the detailed search strategy in each database. Besides, We searched all references in relevant articles and reviews for other eligible studies.

### Inclusion and exclusion criteria

The following eligibility criteria were used in this review:

*Inclusion criteria*:

1. Patients with a known or predicted difficult airway scheduled for elective surgery requiring endotracheal intubation.
2. CSES (COOK Critical Care, Bloomington, IN, USA) was used as the extubation tool;
3. Single-arm observational study;
4. The rate of intubation failure, patients’ tolerance, and evidence of airway injury should be provided.

*Exclusion criteria*:

1. The non-English article was excluded;
2. Reviews, conference abstracts, case reports, letters, and animal trials were excluded from this review;
3. Studies with a sample size of less than ten were excluded;
4. Full text and statistical methods were not available;
5. Literature with duplicate results.

### Data extraction

The characteristics of the included studies are summarized as follows: the authors of the articles, publication year, study location, study design, study site, study population, physical status, type of surgery, predictive criteria of difficult airways, gender, age, and body mass index (BMI). The primary outcome was the intubation success rate, and the secondary outcome was patients’ tolerance and adverse events related to intubation. Information was extracted from eligible studies by two authors independently. Disagreements were resolved by discussion among all authors.

### Quality assessment

The Joanna Briggs Institute (JBI) Critical Appraisal tools for Case Series were used to assess the quality of the risk of bias of the included studies [10]. The assessment indicators are provided in Table 2. “Yes,“ “No,“ and “Unclear” can be used to assess the indicators. There were 10 questions, and a response of “No” to any of the questions negatively impacts the quality of a case series.

### Data analysis

The software R studio, version 4.2.2. was selected to perform the statistical analysis. The continuous data were not normally distributed, so we did a logit transformation when analyzing the data. The Cochrane Q and *I*^2^ statistics were used to test the heterogeneity among all studies. *I*^2^ > 50% indicates the existence of heterogeneity. 0.10 of the p-value or 50% of the I^2^ was considered the critical value of heterogeneity. The fixed and random effects models was used to analyze the homogenous and heterogeneous datasets. We used the DerSimonian-Laird estimator to estimate the between-study variance [11]. Due to the high heterogeneity in the analysis, we use the Arcsine test for publication bias [12]. A P value below 0.05 was regarded as statistically significant. We did a sensitivity analysis with the leave-one-out method to assess the potential confounding effects of intubation success rate, patients’ tolerance, and adverse events related to intubation. According to the possible heterogeneity factors, subgroup analysis was conducted to explore the source of heterogeneity. A linear regression test was performed with the funnel plot to test the publication bias.

## Results

### Study selection and study characteristics

**Fig. 1:**
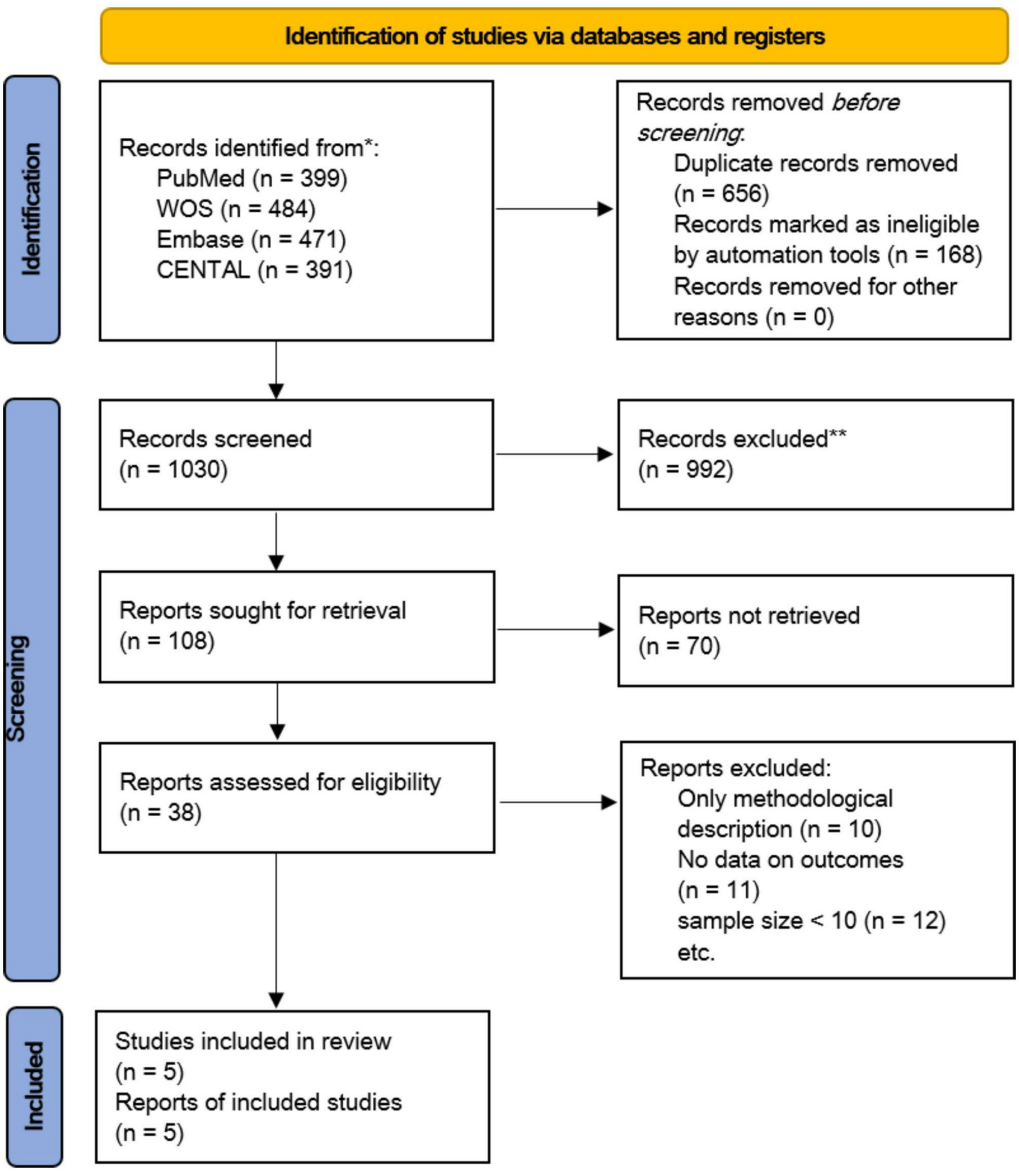
Flowchart of the study selection for the meta-analysis

In sum, 1745 articles were identified in electronic and manual searches. However, 656 articles were excluded for duplication. And 168 articles were marked as ineligible by automation tools, which included reviews, case reports, dissertations, conference papers, chapters in handbooks, and editorials. In addition, 1062 records were excluded after reviewing the title and abstract, and we excluded 33 records after reviewing the full text of 38 articles. Finally, five articles [8, 9, 12–14] were included in this meta-analysis (Fig. 1).

The characteristics of the eligible studies are listed in Table 1. The sample sizes of participants in each study ranged from 20 to 527, and this meta-analysis included 708 difficult airway patients. 4 of the five studies were prospective observational designs, and 1 was a retrospective cohort study. The publication year were ranged from 2005 to 2020. The study site involved Australia (n = 2), the United States (n = 2), and Italy(n = 1). Three single-center studies and two multicentric studies were included. 3 of the five studies provided patients’ preoperative physical status. The proportion of female participants varied significantly between studies (22–60%). 4 of the five studies involved adults, and one targeted pediatric patient. 2 studies reported preoperative BMI in adult patients with a mean of 30.9 and 27.4, respectively. The American Society of Anesthesiologists (ASA) was used in 2 studies to assess preoperative physical status. McManus et al. provided comorbidity status, and high hypertension incidence was observed. 2 studies provided details on the type of surgery. Head and neck surgery is the most common procedure in adult patients with a difficult airway, and cleft palate repair is the most common surgery in children. Cormack and Lehane Grade was used in 2 studies as preoperatively difficult airways predictive criteria [15]. Corso et al. selected 2005 Italian guidelines to define difficult airways [8]. For pediatric patients, difficult intraoperative intubation, airway edema secondary to surgical manipulation, cervical immobility, or instability were considered risk factors for difficult tracheal reintubation.

**Table 1:** Characteristics of enrolled studies for meta-analysis

Author, Year	Enrollment period	Country	Center	Study design	Physical status	Types of surgery	Predictive criteria of difficult airways	Sample size	Female sex - no. (%)	Age - mean (sd)	BMI - kg/m^2^
Corso 2020	Jan 2016 - Dec 2017	Italy	Multicentric	Prospective observational study	ASA score ≥ 3 : 69%	Head and neck surgery: 57 (50%) Orthopedic surgery: 3 (2.6%) Bariatric surgery: 3 (2.6%) Urologic surgery: 6 (5.3%) General surgery: 45 (39.5%)	2005 Italian guidelines and EGRI	114	30 (26.4)	56.0(13.6)	30.9(5.1)
Furyk 2017	May 2015 - Jun 2016	Australian	Single center	Prospective observational trial	ASA score 1 or 2	Elective surgery which required oral endotracheal intubation	C&L: 3 or 4	23	14(60)	52.4 (18.1)	27.4 (4.1)
McLean 2013	Jun 2006 - Oct 2012	US	Single center	Retrospective cohort study	/	Patients undergoing general anesthesia	/	527	/	≥ 18 year old	/
McManus 2018	/	Australia	Single center	Prospective cohort study	Hypertension: 12 Chronic Renal Impairment: 2 Type 2 Diabetes: 2 Asthma: 1 COPD: 1 Obstructive Sleep Apnoea: 1 Arrhythmia: 1	/	C&L 1: 4 C&L 2: 3 C&L 3: 9 C&L 4: 6	23	5(22)	48.7 (17.1)	/
Wise-Faberowski 2005	/	US	Multicentric	Prospective observational trial	/	The most common surgical Procedure: cleft palate repair	Difficult intraoperative intubation; Airway edema secondary to surgical manipulation; Cervical immobility, or instability	20	11 (55%)	114 (75) months	/

ASA: American Society of Anesthesiologists; C&L: Cormack and Lehane Grade; EGRI: El Ganzouri Risk Index

### Study quality

Table 2 provides the included studies’ quality assessment results. 2 of the five studies answered the 10 JBI quality assessment questions. McLean et al. did not describe the patient’s physical status. McManus et al. and Wise-Faberowski et al. did not provide enrollment period, physical status, or types of surgery information; hence the integrity and continuity of the included participant were unclear.

**Table 2:** JBI Critical Appraisal quality assessment for Included studies

Study	(1)	(2)	(3)	(4)	(5)	(6)	(7)	(8)	(9)	(10)
Corso 2020	Y	Y	Y	Y	Y	Y	Y	Y	Y	Y
Furyk 2017	Y	Y	Y	Y	Y	Y	Y	Y	Y	Y
McLean 2013	Y	Y	N	Y	Y	Y	Y	Y	Y	Y
McManus 2018	Y	Y	Y	Un	Un	Y	Y	Y	Y	Y
Wise-Faberowski 2005	Y	Y	Y	Un	Un	Y	Y	Y	Y	Y

*Note*: (1) Were there clear criteria for inclusion in the case series? (2) Was the condition measured in a standard, reliable way for all participants included in the case series? (3) Were valid methods used for identification of the condition for all participants included in the case series? (4) Did the case series have consecutive inclusion of participants? (5) Did the case series have complete inclusion of participants? (6) Was there clear reporting of the demographics of the participants in the study? (7) Was there clear reporting of clinical information of the participants? (8) Were the outcomes or follow up results of cases clearly reported? (9) Was there clear reporting of the presenting site(s)/clinic(s) demographic information? (10) Was statistical analysis appropriate?

Y: Yes; N: No; Un: Unclear

### Primary outcome

**Fig. 2:**
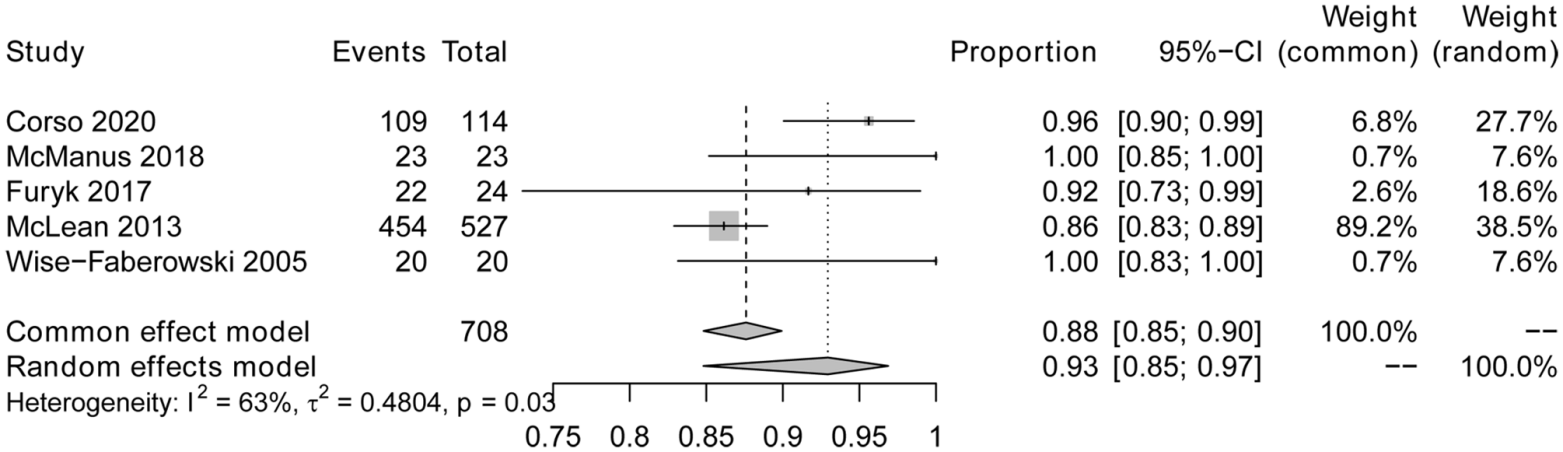
Forest plot for CSES success rate

**Fig. 3:**
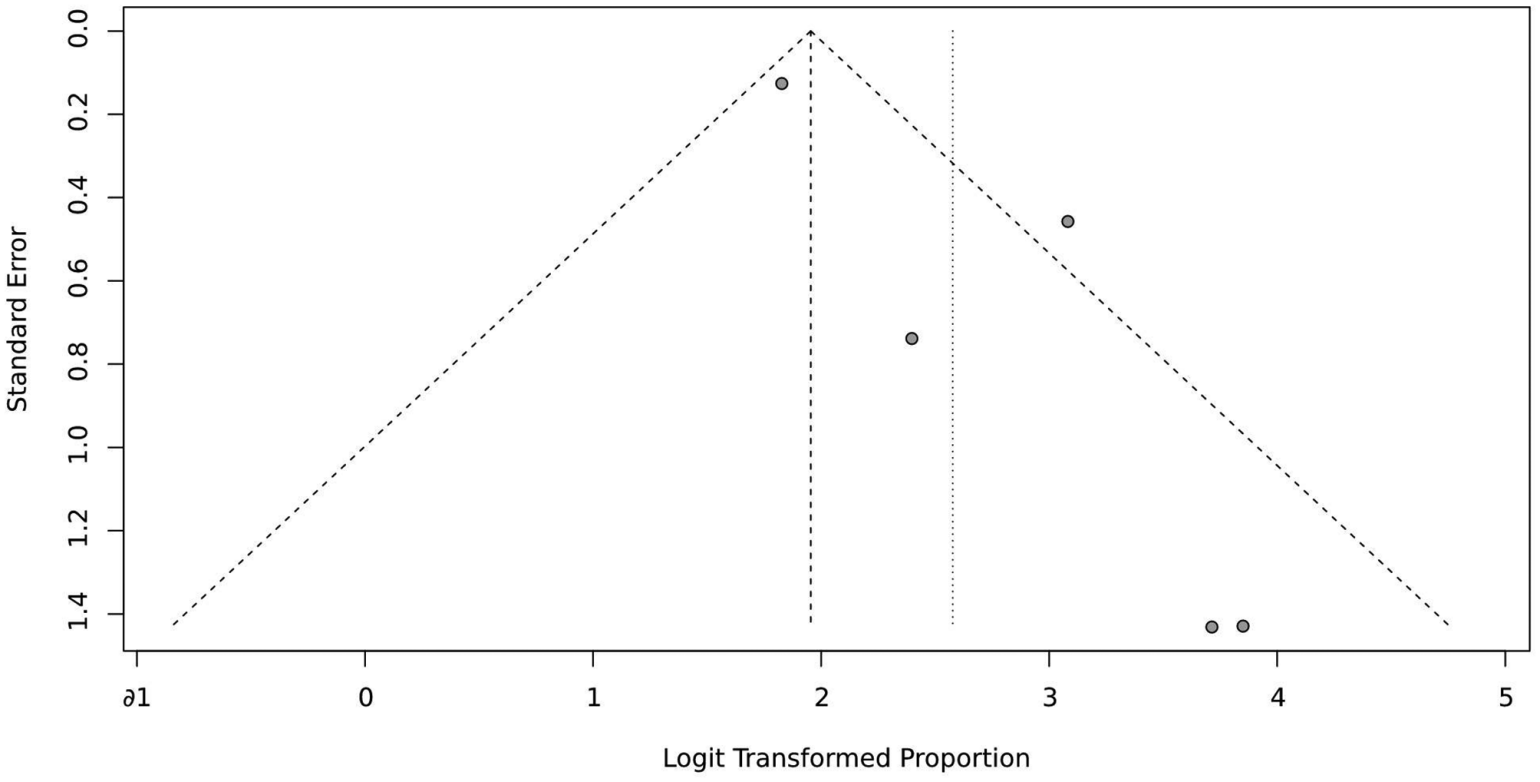
Funnel plot for CSES success rate

**Fig. 4:**
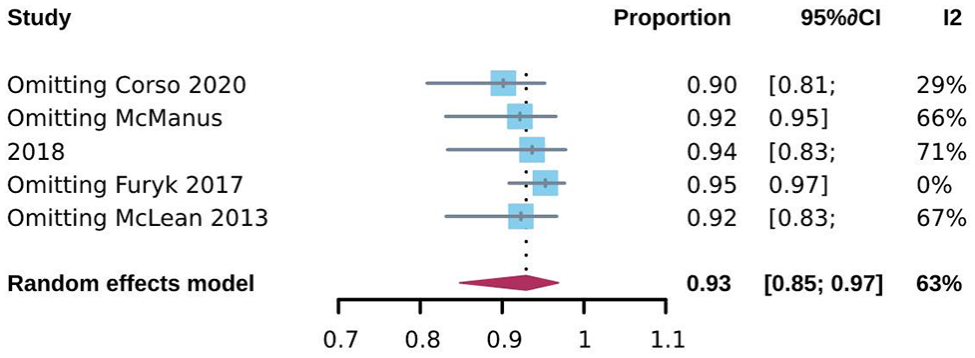
Sensitivity analysis for CSES success rate

To investigate CSES efficacy for difficult airways, we analyzed the pooled overall CSES clinical success rate as the primary outcome. Five included studies involving 708 patients who achieved a response after CSES. Heterogeneity testing showed that I2 = 63% and p = 0.03. The statistical meta-analysis using the random-effects model showed that the overall clinical success rate was 93% (95% CI: 85%, 97%) (Fig. 2). High publication bias was demonstrated by funnel plot (Fig. 3). Linear regression test was performed among four studies with age and female ratio information, and the t-test result showed that neither of the two covariables had any effect on the success rate of CSES (t = 2.99, p = 0.058) (Table 3). In addition, excluding any of the five studies, the combined results of the remaining four studies were consistent with the original ones, indicating the stability of the outcome (Fig. 4).

**Table 3:** Linear regression test of funnel plot asymmetry

Outcome	t	df	p-value
SES success	2.99	3	0.058
SES intolerable	0.07	1	0.9546

### Secondary outcomes

**Fig. 5:**
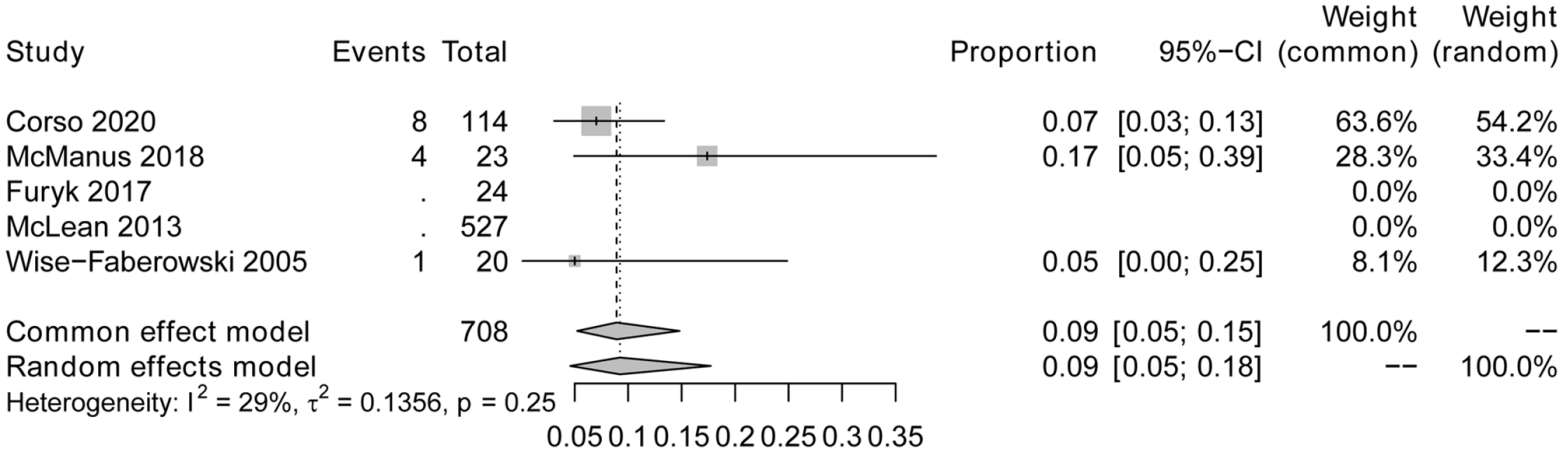
Forest plot for CSES intoletable rate

**Fig. 6:**
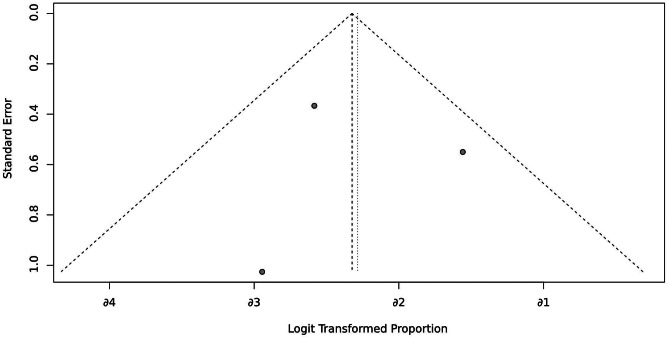
Funnel plot for CSES intolerable rate

**Fig. 7:**
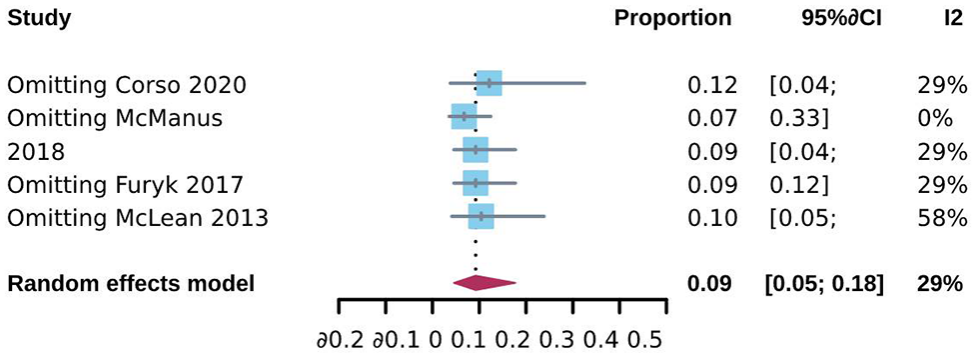
Sensitivity analysis for CSES intolerable rate

Three studies recorded the CSES intolerable rate as the indicator of CSES tolerance. The pooled result analyzed by the fixed-effects model showed that the CSES intolerable rate was 9% (95% CI: 5%, 18%). Heterogeneity testing showed that I2 = 29% and p = 0.25 (Fig. 5). Potential publication bias was observed from the funnel plot (Fig. 6). Linear regression test was based on age and female ratio, and the t-test result showed that neither of the two covariables had any effect on CSES intolerable rate (t = 0.07, p = 0.95) (Table 3). The sensitivity analysis results proved the results’ robustness (Fig. 7).

### Subgroup analysis

**Fig. 8:**
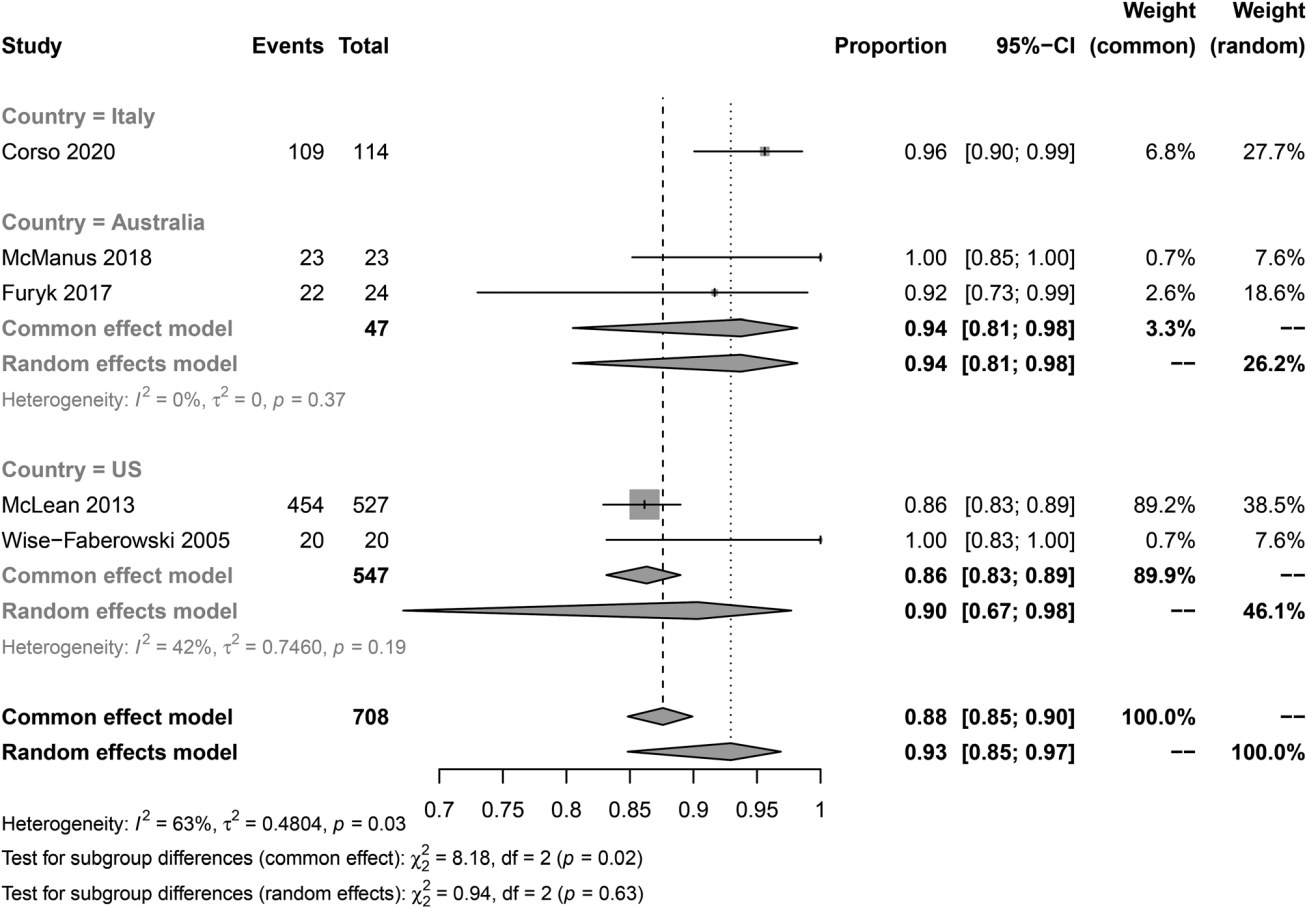
Subgroup analysis for study location

**Fig. 9:**
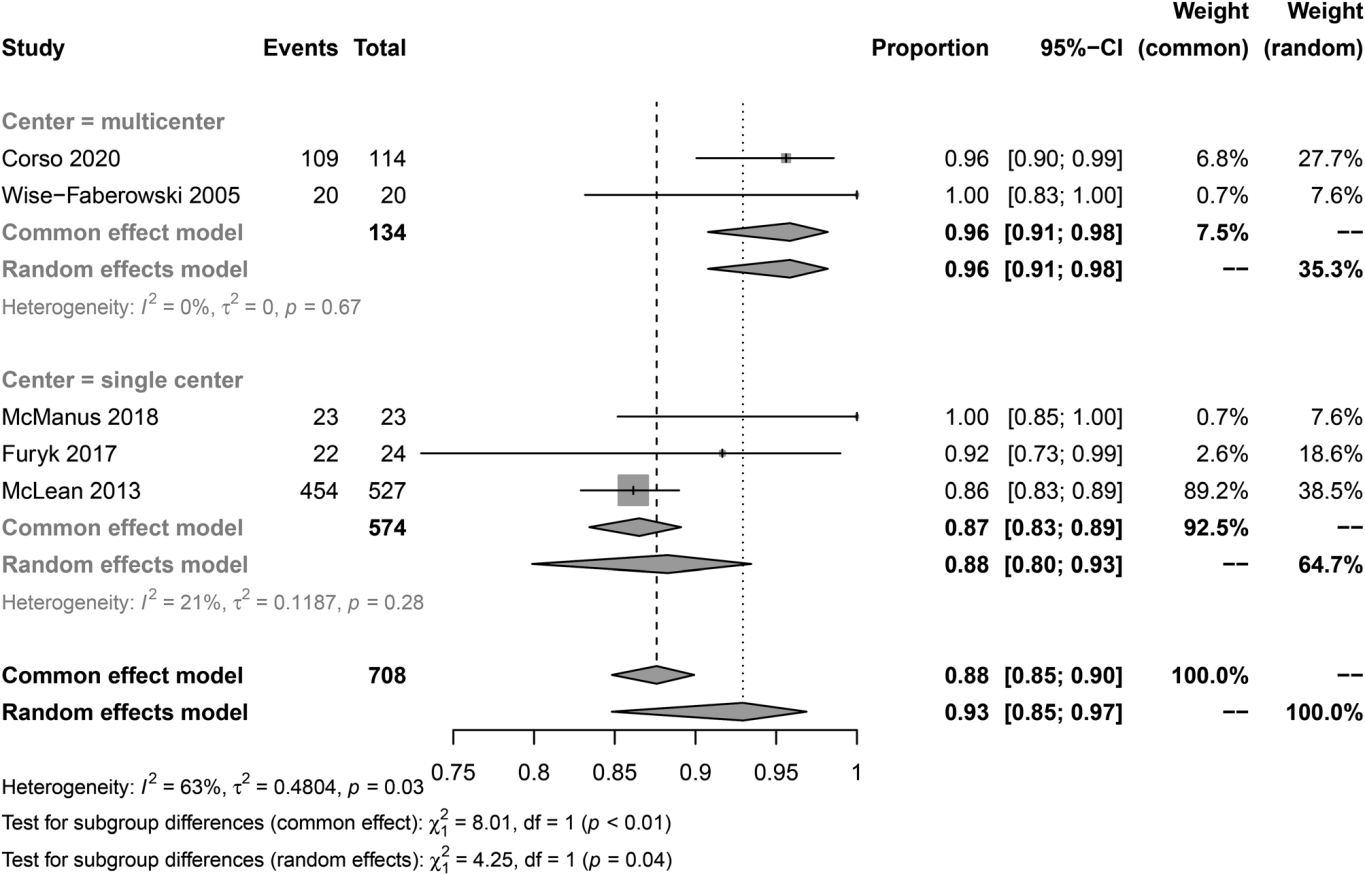
Subgroup analysis for study center

**Fig. 10:**
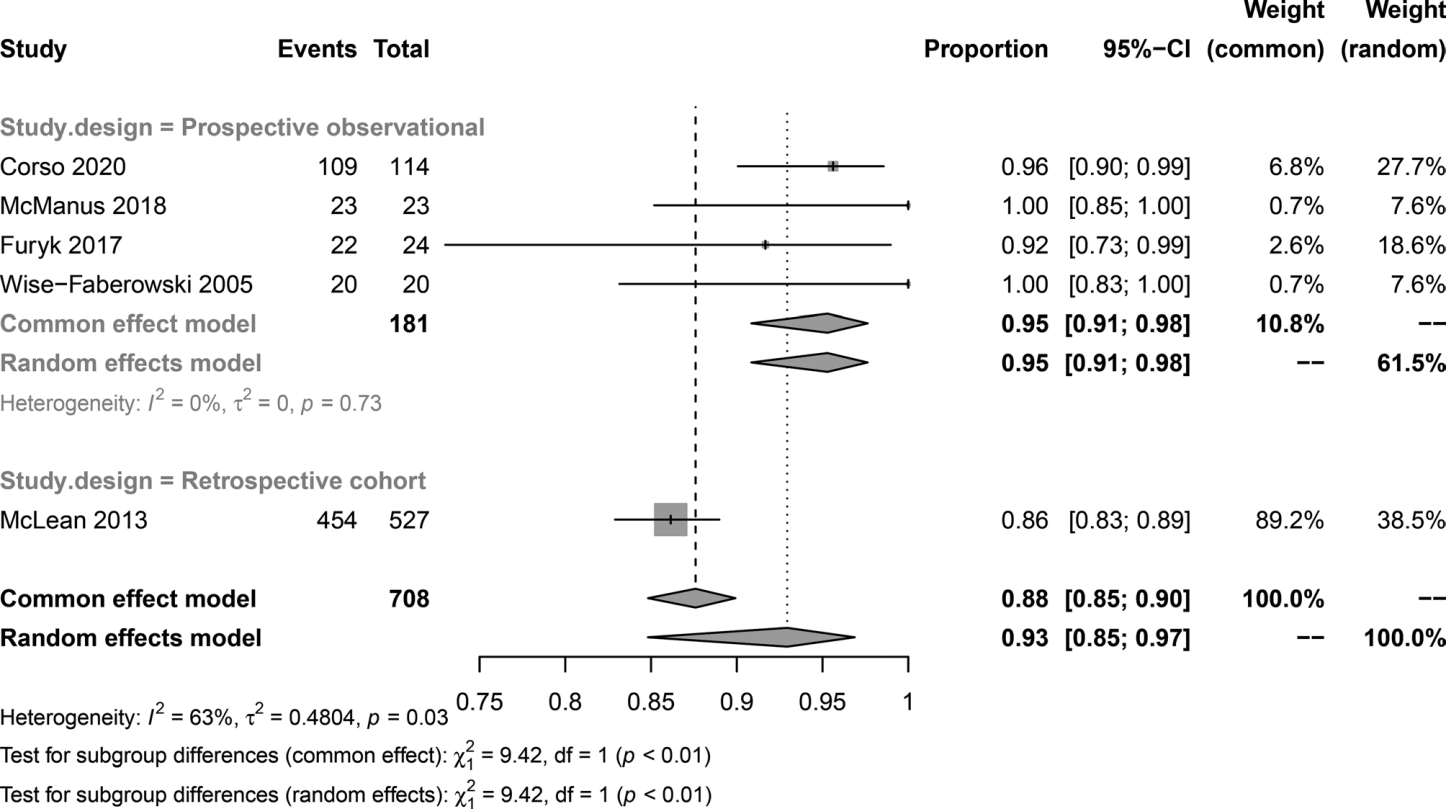
Subgroupanalysis for study design

The subgroup analysis of CSES clinical success rate showed that the pooled overall clinical success rates in Italian patients, Australian patients, and US patients were 96% (95% CI: 90%, 99%), 94% (95% CI: 81%, 98%), and 90% (95% CI: 67–98%) respectively. No significant difference was observed between subgroups (p = 0.63) (Fig. 8). The pooled CSES clinical success rates in multicentric studies were 96% (95% CI: 91%, 98%) and 87% (95% CI: 83%, 89%) in single-center studies. A significant difference was observed between the two subgroups (p = 0.04) (Fig. 9). The pooled CSES success rates in prospective studies were 95% (95% CI: 91%, 98%) and 86% (95% CI: 83%, 89%) in retrospective studies. A significant difference was observed between the two subgroups (p < 0.01) (Fig. 10).

### Incidence rate of complications

**Fig. 11:**
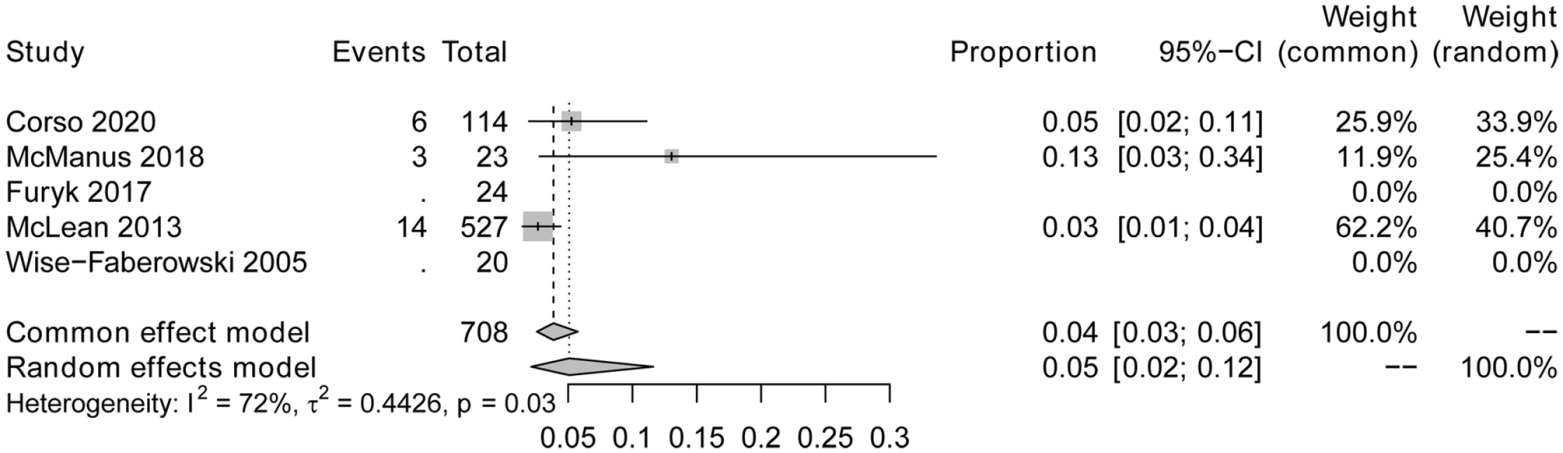
Forest plot for complication incidence

**Fig. 12:**
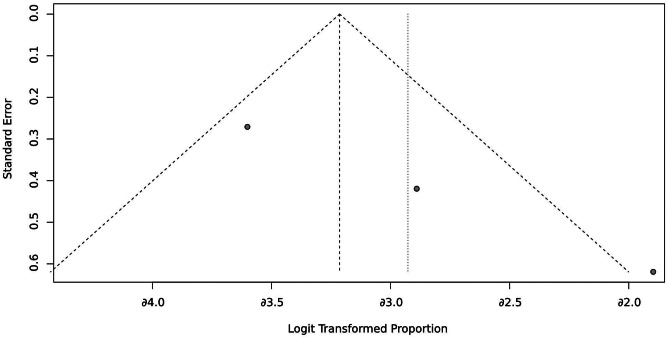
Funnel plot for Complication incidence

**Fig. 13:**
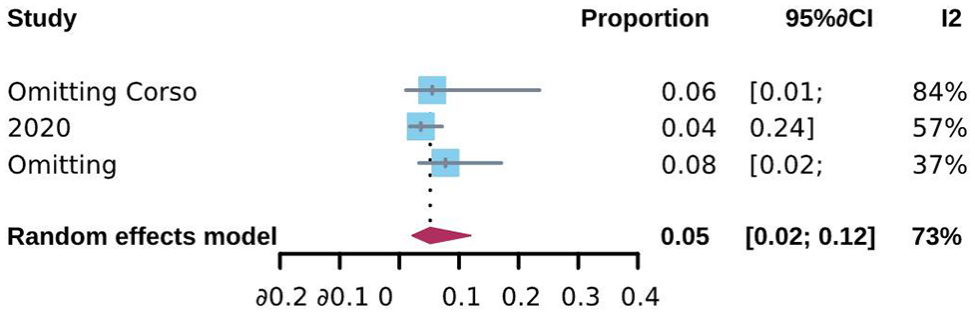
Sensitivity analysis for Complication incidence

The pooled incidence rate of complications was analyzed to investigate the safety and complications of CSES for difficult airway patients. A total of 3 studies reported complications and involved 664 patients. 22 patients had adverse reactions during and after CSES. The heterogeneity testing showed that I^2^ = 72%, p = 0.03, indicating heterogeneity among the studies. The statistical meta-analysis with the random-effects model showed that the pooled overall incidence rate of complications was 5% (95% CI: 2%, 12%) (Fig. 11). No publication bias was observed, and sensitivity analysis proved the stability (Figs. 12, 13). In addition, the complications reported by the three studies included airway injury, hypoxemia, lip trauma, pneumothorax, and intolerable symptoms during CSES. Among these, pneumothorax, reported by McLean et al., accounted for the highest proportion of complications.

### Systematic review for eligible case report

During the literature search, it was found that except for the literature eligible for meta-analysis, a large number of case reports recorded the effect of CSES as an intubation tool in difficult airway patients. Therefore, we integrated the results of these case reports in a systematic review as additional evidence of the clinical effectiveness of CSES in patients with difficult airways.

In sum, 13 case reports were identified through title and abstract screening. However, 6 case reports were excluded because the full text was unavailable. The characteristics and main outcomes of the included 7 case reports [16–22] were extracted in Table 4. The publication year ranged from 1997 to 2013. Three were from the United States, two were from Korea, one was from Lebanon, and one was from Japan. One of the seven patients with difficult airways was a pediatric patient aged 33 days. The remaining six patients are adults, including one female and five males. Seven studies have documented the successful use of CSES to achieve safe intubation and extubation without complications during surgery in 7 patients with different conditions. Intolerance of CSES was not observed in the case reports. Baraka et al. reported an increased risk of barotrauma and tension pneumothorax due to intermittent oxygen injection (50 psi) through the airway exchange catheter lumen.

**Table 4:** Characteristics of enrolled studies for systematic review

Author, Year	Country	Sample size	Gender	Age	Physical status	Types of surgery	Predictive criteria of difficult airways	CSES result	Tolerance	Complications
Choi 2013	Korea	1	Male	33-day	C-section Pierre Robin syndrome	Glossopexy surgery	Difficulty in maintaining patent upper airway	Success	/	N
Baraka 1999	Baraka	1	Male	45-year-old	Overweight	Laparoscopic cholecystectomy	Several failed intubation attempt	Success	/	Potential pneumothorax risk
Gruenbaum 2012	US	1	Female	32-year-old	180 cm height	Emergency cesarean section	Several failed intubation attempts	Success	/	N
Hartmannsgruber 1997	US	1	Male	77-year-old	Trauma, fracture, and rotary subluxation	Occipital-cervical fusion	Several failed intubation attempts	Success	/	/
Moyers 2002	US	1	Male	43-year-old	Morbidly obese	Enlarged thyroid gland	Significant tracheal deviation secondary to an enlarged thyroid gland	Success	/	/
Nomoto 2003	Japan	1	Male	68-year-old	Cancer	Intractable and progressive dyspnea	Lower tracheal stenosis due to neoplastic invasion	Success	/	N
Kim 2012	Korea	1	Male	31-year-old	Laryngeal papilloma	Removal of the papilloma	/	Success	/	N

The type of surgery and the cause of the difficult airway in the pediatric patients in the case report and meta-analysis were both lip-tongue-related malformations [16]. The difficulty of tracheal intubation was aggravated by obesity in both overweight patients [17, 20]. The cause of airway difficulties in 2 tumor patients was tracheal stenosis caused by tumor invasion, and both patients were accompanied by dyspnea [21, 22]. An elderly patient (77-year-old) with severe injuries was treated with CSES due to repeated failed intubation attempts [19]. Gruenbaum et al. reported on a patient who had repeatedly failed to intubate due to her height (180 cm) and insufficient length of the Aintree intubating catheter. A longer (100 cm) exchange catheter was replaced, and the upper endotracheal catheter successfully entered the trachea and completed the C-section  [18].

## Discussion

This single-arm systematic review and meta-analysis is the first study to analyze the success rate and safety of CSES as an extubation tool in patients with a difficult airway. We included 714 difficult airway patients using CSES as an extubation tool. Several significant findings were observed from the meta-analysis results. As the primary and secondary outcome, CSES has achieved a high clinical success rate in adult and pediatric patients with different physical conditions and types of surgery. All original studies and meta-analyses confirmed a remarkably high tolerance rate and low overall complication rate. From the results of subgroup analysis and linear regression analysis, it can be inferred that the study’s higher heterogeneity and potential publication bias may result from the differences in the number of centers and study design of the original study. Loss or displacement of guidewire due to difficulty fixing is the main cause of CSES extubation failure and intolerance. Although the complication rate appears acceptable, CSES may increase the user’s risk of airway injury or pneumothorax.

CSES is a kind of hollow semi-rigid oxygen supply catheter, which is used in tube exchange or extubation tests because its catheter tip is blunt, which reduces the risk of tracheobronchial injury or lung tear [23]. Unlike conventional jet-ventilated catheters, airway management may be easier and safer for anesthesiologists because CSESs can be used for endotracheal intubation through a catheter as a guide needle, can be connected with a typical circuit, an injector for high-frequency jet ventilation, or monitor end-tidal CO2 adapter through a RAPI-FIT® adapter [24]. Different types and sizes of CSESs are produced, and the anesthesiologist can select the appropriate CSESs for the case for effective airway management during anesthesia. In addition, in terms of implantation mode, studies have evaluated the dislocation rate of transoral or intranasal AECs and have not shown significant differences between transoral and intranasal approaches.

Subgroup analysis and linear regression explain some of the reasons for the heterogeneity of the original study through quantitative data. Usually, well-designed multicentre studies may have more rigorous requirements for the execution of CSES operations, and the data integrity may be higher in prospective studies. Thus, CSES show higher clinical success rates in these studies. In addition, the difference in the definition of the clinical success rate of CSES is also the main cause of heterogeneity. Three of the included studies defined CSES success as successful intubation. McLean et al. described CSES success as complete trachea tube change on time. And Corso et al. defind CSES success as able to reintubate. Besides, none of the seven patients in the systematic review had reintubation. The original intention of staged extubation of CSES is to ensure the success rate and safety of extubation and reintubation for patients with airway difficulties to the greatest extent. However, the current result can only prove CSES’s high intubation success rate. Corso et al. reported that the CSES reintubation failure rate in difficult airway patients was 33.3%, which remains quite more elevated than the results from the study also recruited regular intubation patients (25%) [14]. Considering the high reintubation and low reintubation success rates in patients with a difficult airway, future studies should pay more attention to the success rate and safety of CSES used for reintubation.

Current studies of CSES have a relatively small sample size, so overall complication reporting rates are low. However, the increased risk of airway injury and pneumothorax due to CSES use is a common problem reported by multiple studies. A recent review showed that 80% (95% CI, 69–88) of patients with airway intubation had no injury. Oedema is the most common mild injury, with prevalence ranging from 9 to 84%. Vocal cord hematoma is the most common moderate injury, with a prevalence of 4% (95% CI, 2–10). Overall, laryngeal injuries from short surgical intubations are common and usually mild. Screening for speech difficulties and dysphagia after extubation may help identify injuries [25]. For pneumothorax caused by CSES. Case report studies speculate that the presence of endotracheal exchangers can significantly reduce the cross-sectional area and impairs passive exhalation, leading to air retention and consequent barotrauma. In addition, the tip of the exchange catheter may wedge into the bronchi, thus preventing air escape [17]. In response to this hypothesis, combined barometric trauma can be reduced by reducing airway pressure during jet ventilation, providing longer exhalation times, and selecting an appropriately sized exchange catheter, all of which prevent air retention [26].

Based on the available evidence, the experience we can share is that unrecognized oesophagal intubation events should be identified and prevented among the high reintubation situation in difficult airway patients. Recommendations such as exhaled carbon dioxide monitoring and pulse oxygen saturation measurement, video laryngoscope use, detecting the presence of “persistent exhaled carbon dioxide”; carry out cross-professional education programs can be used by all airway professionals in the prevention of unrecognized oesophagal intubations [27]. At the same time, although relatively safe and effective auxiliary reintubation tools such as CSES have entered clinical application, considering the risk of airway injury and intubation failure caused by reintubation, the venturi mask or even better high-flow nasal oxygen cannula (HFNC) may be the better choices for inexperienced airway practitioners. It has been proved that both venturi masks and HFNC may help icu extubation patients achieve better oxygenation [28]. High Flow nasal oxygen cannula may also achieve even lower rates of intubation [29]. This is beneficial to the oxygen supply of patients after extubation and the formulation of a reintubation plan.

In conclusion, CSES is a safer and more feasible intubation tool for patients with clinical airway difficulties. However, compared with tool selection, the importance of developing a safe strategy for intubation and extubation before surgery, as well as the anesthesiologist’s proficiency and familiarity with the guidelines, has been emphasized in all studies.

### Limitations

This study has some inventive new findings and some limits. First, the included number and the sample size of this meta-analysis were small, and the selection of patients in the original study had significant differences in their characteristics, preoperative physical conditions, and type of surgeries. Besides, due to the absence of patients’ basic information in some of the original studies, it is difficult to determine the source of inter-study heterogeneity. Second, the lack of a control arm. Since the clinical efficacy and safety studies of CSES are still at a relatively preliminary stage, the number of relevant works of literature is small, and no RCTS have been published. This meta-analysis based on single-arm studies is intended to point out the direction for future research. The caveat to establishing controlled trials in these settings is that it is difficult to recruit patients due to the severity of their condition in patients with difficult airways requiring surgery, which will result in underpowered studies. In addition, conducting controlled trials under such painful conditions may raise ethical questions related to surgical success rates and postoperative complications.

## Conclusions

This meta-analysis suggested that CSES have achieved a high clinical success rate in adult and pediatric patients with different physical conditions and types of surgery. All original studies and meta-analyses confirmed a remarkably high tolerance rate and low overall complication rate. However, regardless of the tools chosen, a personalized, safe intubation strategy and a highly qualified anesthesiologist should be considered as the fundamental guarantee of a high clinical success rate. Future studies should also focus on the success rate of reintubation using CSES in patients with airway difficulties.

## Electronic supplementary material

Below is the link to the electronic supplementary material.

Supplementary Material 1

## Data Availability

All data generated or analysed during this study are included in this published article and its supplementary information files.

## Abbreviations

AECs: Airway exchange catheters
JBI: Joanna Briggs Institute
ASA: American Society of Anesthesiologists
