# Supplementary material for: Efficacy and safety of Cook staged Extubation Set in patients with difficult airway: a systematic review and meta-analysis
Source: BMC Anesthesiol. 2023 Jul 7;23:232. doi: 10.1186/s12871-023-02191-0 (PMC10327169; doi:10.1186/s12871-023-02191-0)
Supplement: Supplementary file 1 — Supplementary Material 1 [file 12871_2023_2191_MOESM1_ESM.docx]

**Search strategy of PubMed**

| NO. | Search Details | Results |
| --- | --- | --- |
| #10 | #8 AND #9 | 5,249 |
| #9 | #1 or #2 or #3 or #4 or #5 or #6 or #7 | 10,093 |
| #8 | ("intubation, intratracheal"[MeSH Terms] OR ("intubation"[All Fields] AND "intratracheal"[All Fields]) OR "intratracheal intubation"[All Fields] OR ("endotracheal"[All Fields] AND "intubation"[All Fields]) OR "endotracheal intubation"[All Fields]) OR (("airway"[All Fields] OR "airway s"[All Fields] OR "airways"[All Fields]) AND ("airway extubation"[MeSH Terms] OR ("airway"[All Fields] AND "extubation"[All Fields]) OR "airway extubation"[All Fields] OR "extubated"[All Fields] OR "extubation"[All Fields] OR "extubations"[All Fields] OR "extubate"[All Fields] OR "extubating"[All Fields])) | 52,720 |
| #7 | ("bailey"[All Fields] OR "bailey s"[All Fields]) AND ("maneuver"[All Fields] OR "maneuvered"[All Fields] OR "maneuvering"[All Fields] OR "maneuverings"[All Fields] OR "maneuvers"[All Fields] OR "manoeuvrability"[All Fields] OR "manoeuvrable"[All Fields] OR "manoeuvre"[All Fields] OR "manoeuvred"[All Fields] OR "manoeuvres"[All Fields] OR "manoeuvring"[All Fields]) | 35 |
| #6 | "airway extubation"[MeSH Terms] OR ("airway"[All Fields] AND "extubation"[All Fields]) OR "airway extubation"[All Fields] OR ("tracheal"[All Fields] AND "extubation"[All Fields]) OR "tracheal extubation"[All Fields] | 5,894 |
| #5 | ("trachea"[MeSH Terms] OR "trachea"[All Fields] OR "tracheal"[All Fields] OR "tracheally"[All Fields]) AND "tube"[All Fields] AND ("exchangable"[All Fields] OR "exchange"[All Fields] OR "exchangeabilities"[All Fields] OR "exchangeability"[All Fields] OR "exchangeable"[All Fields] OR "exchanged"[All Fields] OR "exchanger"[All Fields] OR "exchanger s"[All Fields] OR "exchangers"[All Fields] OR "exchanges"[All Fields] OR "exchanging"[All Fields]) AND ("catheter s"[All Fields] OR "catheters"[MeSH Terms] OR "catheters"[All Fields] OR "catheter"[All Fields]) | 94 |
| #4 | ("stage"[All Fields] OR "staged"[All Fields] OR "stages"[All Fields] OR "staging"[All Fields] OR "stagings"[All Fields]) AND ("airway extubation"[MeSH Terms] OR ("airway"[All Fields] AND "extubation"[All Fields]) OR "airway extubation"[All Fields] OR "extubated"[All Fields] OR "extubation"[All Fields] OR "extubations"[All Fields] OR "extubate"[All Fields] OR "extubating"[All Fields]) AND "kit"[All Fields] | 3 |
| #3 | ("cooking"[MeSH Terms] OR "cooking"[All Fields] OR "cook"[All Fields]) AND ("stage"[All Fields] OR "staged"[All Fields] OR "stages"[All Fields] OR "staging"[All Fields] OR "stagings"[All Fields]) AND ("airway extubation"[MeSH Terms] OR ("airway"[All Fields] AND "extubation"[All Fields]) OR "airway extubation"[All Fields] OR "extubated"[All Fields] OR "extubation"[All Fields] OR "extubations"[All Fields] OR "extubate"[All Fields] OR "extubating"[All Fields]) AND ("bone wires"[MeSH Terms] OR ("bone"[All Fields] AND "wires"[All Fields]) OR "bone wires"[All Fields] OR "wire"[All Fields]) | 4 |
| #2 | ("stage"[All Fields] OR "staged"[All Fields] OR "stages"[All Fields] OR "staging"[All Fields] OR "stagings"[All Fields]) AND ("airway extubation"[MeSH Terms] OR ("airway"[All Fields] AND "extubation"[All Fields]) OR "airway extubation"[All Fields] OR "extubated"[All Fields] OR "extubation"[All Fields] OR "extubations"[All Fields] OR "extubate"[All Fields] OR "extubating"[All Fields]) | 632 |
| #1 | (("airway"[All Fields] OR "airway s"[All Fields] OR "airways"[All Fields]) AND ("exchangable"[All Fields] OR "exchange"[All Fields] OR "exchangeabilities"[All Fields] OR "exchangeability"[All Fields] OR "exchangeable"[All Fields] OR "exchanged"[All Fields] OR "exchanger"[All Fields] OR "exchanger s"[All Fields] OR "exchangers"[All Fields] OR "exchanges"[All Fields] OR "exchanging"[All Fields]) AND ("catheter s"[All Fields] OR "catheters"[MeSH Terms] OR "catheters"[All Fields] OR "catheter"[All Fields])) OR ("AEC"[All Fields]) | 3,755 |

**Search strategy of Web of Science**

| NO. | Search Details | Results |
| --- | --- | --- |
| #10 | #8 AND #9 | 3219 |
| #9 | #1 or #2 or #3 or #4 or #5 or #6 or #7 | 11988 |
| #8 | (( TS="intubation intratracheal" OR TS="intratracheal intubation" OR TS="endotracheal intubation") OR (TS="airway" OR TS="airway s" OR TS="airways")) AND (TS="airway extubation" OR TS="extubated" OR TS="extubation" OR TS="extubations" OR TS="extubate" OR TS="extubating") | 8039 |
| #7 | (TS="bailey" OR TS= "bailey s") AND ("maneuver" OR TS= "maneuvered" OR TS= "maneuvering" OR TS= "maneuverings" OR TS= "maneuvers" OR TS= "manoeuvrability" OR TS= "manoeuvrable" OR TS= "manoeuvre" OR TS= "manoeuvred" OR TS= "manoeuvres" OR TS= "manoeuvring") | 7 |
| #6 | TS="airway extubation" OR TS="tracheal extubation" | 3801 |
| #5 | (TS="trachea" OR TS= "tracheal" OR TS= "tracheally") AND TS="tube" AND (TS="exchangable" OR TS="exchange" OR TS="exchangeabilities" OR TS="exchangeability" OR TS="exchangeable" OR TS="exchanged" OR TS="exchanger" OR TS="exchanger s" OR TS="exchangers" OR TS="exchanges" OR TS="exchanging") AND (TS="catheter s" OR TS="catheters" OR TS="catheter") | 137 |
| #4 | (TS="stage" OR TS="staged" OR TS="stages" OR TS="staging" OR TS="stagings") AND (TS="airway extubation" OR TS="extubated" OR TS="extubation" OR TS="extubations" OR TS="extubate" OR TS="extubating") AND TS="kit" | 3 |
| #3 | (TS="cooking" OR TS="cook") AND (TS="stage" OR TS="staged" OR TS="stages" OR TS="staging" OR TS="stagings") AND (TS="airway extubation" OR TS="extubated" OR TS="extubation" OR TS="extubations" OR TS="extubate" OR TS="extubating") AND (TS="bone wires" OR TS="wire") | 4 |
| #2 | (TS="stage" OR TS="staged" OR TS="stages" OR TS="staging" OR TS="stagings") AND (TS="airway extubation" OR TS="extubated" OR TS="extubation" OR TS="extubations" OR TS="extubate" OR TS="extubating") | 738 |
| #1 | ((TS="airway" OR TS="airway s" OR TS="airways") AND (TS="exchangable" OR TS="exchange" OR TS="exchangeabilities" OR TS="exchangeability" OR TS="exchangeable" OR TS="exchanged" OR TS="exchanger" OR TS="exchanger s" OR TS="exchangers" OR TS="exchanges" OR TS="exchanging") AND (TS="catheter s" OR TS="catheters" OR TS="catheters" OR TS="catheter")) OR (TS="AEC") | 7581 |

**Search strategy of EMBASE**

| No. | Query | Results |
| --- | --- | --- |
| #10 | #8 AND #9 | 471 |
| #9 | #1 or #2 or #3 or #4 or #5 or #6 or #7 | 6,403 |
| #8 | (‘intubation, intratracheal’:ti,ab,kw OR (‘intubation’:ti,ab,kw AND ‘intratracheal’:ti,ab,kw) OR ‘intratracheal intubation’:ti,ab,kw OR (‘endotracheal’:ti,ab,kw AND ‘intubation’:ti,ab,kw) OR ‘endotracheal intubation’:ti,ab,kw) OR ((‘airway’ OR ‘airway s’:ti,ab,kw OR ‘airways’:ti,ab,kw) AND (‘airway extubation’:ti,ab,kw OR (‘airway’:ti,ab,kw AND ‘extubation’:ti,ab,kw) OR ‘airway extubation’:ti,ab,kw OR ‘extubated’:ti,ab,kw OR ‘extubation’:ti,ab,kw OR ‘extubations’:ti,ab,kw OR ‘extubate’:ti,ab,kw OR ‘extubating’:ti,ab,kw)) |  |
| #7 | ('bailey':ti,ab,kw OR 'bailey s':ti,ab,kw) AND ('maneuver':ti,ab,kw OR 'maneuvered':ti,ab,kw OR 'maneuvering':ti,ab,kw OR 'maneuverings':ti,ab,kw OR 'maneuvers':ti,ab,kw OR 'manoeuvrability':ti,ab,kw OR 'manoeuvrable':ti,ab,kw OR 'manoeuvre':ti,ab,kw OR 'manoeuvred':ti,ab,kw OR 'manoeuvres':ti,ab,kw OR 'manoeuvring':ti,ab,kw) |  |
| #6 | 'airway extubation':ti,ab,kw OR 'tracheal extubation':ti,ab,kw |  |
| #5 | ('trachea':ti,ab,kw OR 'tracheal':ti,ab,kw OR 'tracheally':ti,ab,kw) AND 'tube':ti,ab,kw AND ('exchangable':ti,ab,kw OR 'exchange':ti,ab,kw OR 'exchangeabilities':ti,ab,kw OR 'exchangeability':ti,ab,kw OR 'exchangeable':ti,ab,kw OR 'exchanged':ti,ab,kw OR 'exchanger':ti,ab,kw OR 'exchanger s':ti,ab,kw OR 'exchangers':ti,ab,kw OR 'exchanges':ti,ab,kw OR 'exchanging':ti,ab,kw) AND ('catheter s':ti,ab,kw OR 'catheters':ti,ab,kw OR 'catheter':ti,ab,kw) |  |
| #4 | ('stage':ti,ab,kw OR 'staged':ti,ab,kw OR 'stages':ti,ab,kw OR 'staging':ti,ab,kw OR 'stagings':ti,ab,kw) AND ('airway extubation':ti,ab,kw OR 'extubated':ti,ab,kw OR 'extubation':ti,ab,kw OR 'extubations':ti,ab,kw OR 'extubate':ti,ab,kw OR 'extubating':ti,ab,kw) AND 'kit':ti,ab,kw |  |
| #3 | ('cooking':ti,ab,kw OR 'cook':ti,ab,kw) AND ('stage':ti,ab,kw OR 'staged':ti,ab,kw OR 'stages':ti,ab,kw OR 'staging':ti,ab,kw OR 'stagings':ti,ab,kw) AND ('airway extubation':ti,ab,kw OR 'extubated':ti,ab,kw OR 'extubation':ti,ab,kw OR 'extubations':ti,ab,kw OR 'extubate':ti,ab,kw OR 'extubating':ti,ab,kw) AND ('bone wires':ti,ab,kw OR 'wire':ti,ab,kw) | 26,487 |
| #2 | ('stage':ti,ab,kw OR 'staged':ti,ab,kw OR 'stages':ti,ab,kw OR 'staging':ti,ab,kw OR 'stagings':ti,ab,kw) AND ('airway':ti,ab,kw AND 'extubation':ti,ab,kw OR 'airway extubation':ti,ab,kw OR 'extubated':ti,ab,kw OR 'extubation':ti,ab,kw OR 'extubations':ti,ab,kw OR 'extubate':ti,ab,kw OR 'extubating':ti,ab,kw) | 1,413 |
| #1 | ((‘airway’:ti,ab,kw OR ‘airway s’:ti,ab,kw OR ‘airways’:ti,ab,kw) AND (‘exchangable’:ti,ab,kw OR ‘exchange’:ti,ab,kw OR ‘exchangeabilities’:ti,ab,kw OR ‘exchangeability’:ti,ab,kw OR ‘exchangeable’:ti,ab,kw OR ‘exchanged’:ti,ab,kw OR ‘exchanger’:ti,ab,kw OR ‘exchanger s’:ti,ab,kw OR ‘exchangers’:ti,ab,kw OR ‘exchanges’:ti,ab,kw OR ‘exchanging’:ti,ab,kw) AND (‘catheter s’:ti,ab,kw OR ‘catheters’:ti,ab,kw OR ‘catheters’:ti,ab,kw OR ‘catheter’:ti,ab,kw)) OR (‘AEC’:ti,ab,kw) | 5,000 |

**Search strategy of Cochrane Controlled Register of Trials (****CENTAL)**

| NO. | Search deatiles | Hits |
| --- | --- | --- |
| #10 | #8 AND #9 | 1,017 |
| #9 | #1 or #2 or #3 or #4 or #5 or #6 or #7 | 45,041 |
| #8 | ("intubation, intratracheal" OR ("intubation" AND "intratracheal") OR "intratracheal intubation" OR ("endotracheal" AND "intubation") OR "endotracheal intubation") OR (("airway" OR "airway s" OR "airways") AND ("airway extubation" OR ("airway" AND "extubation") OR "airway extubation" OR "extubated" OR "extubation" OR "extubations" OR "extubate" OR "extubating")) | 12,357 |
| #7 | ("bailey" OR "bailey s") AND ("maneuver" OR "maneuvered" OR "maneuvering" OR "maneuverings" OR "maneuvers" OR "manoeuvrability" OR "manoeuvrable" OR "manoeuvre" OR "manoeuvred" OR "manoeuvres" OR "manoeuvring") | 22 |
| #6 | "airway extubation" OR "tracheal extubation" | 1,156 |
| #5 | ("trachea" OR "tracheal" OR "tracheally") AND "tube" AND ("exchangable" OR "exchange" OR "exchangeabilities" OR "exchangeability" OR "exchangeable" OR "exchanged" OR "exchanger" OR "exchanger s" OR "exchangers" OR "exchanges" OR "exchanging") AND ("catheter s" OR "catheters" OR "catheter") | 33 |
| #4 | ("stage" OR "staged" OR "stages" OR "staging" OR "stagings") AND ("airway extubation" OR "extubated" OR "extubation" OR "extubations" OR "extubate" OR "extubating") AND "kit" | 1 |
| #3 | ("cooking" OR "cook") AND ("stage" OR "staged" OR "stages" OR "staging" OR "stagings") AND ("airway extubation" OR "extubated" OR "extubation" OR "extubations" OR "extubate" OR "extubating") AND ("bone wires" OR "wire") | 4 |
| #2 | ("stage" OR "staged" OR "stages" OR "staging" OR "stagings") AND ("airway extubation" OR ("airway" AND "extubation") OR "airway extubation" OR "extubated" OR "extubation" OR "extubations" OR "extubate" OR "extubating") | 421 |
| #1 | (("airway" OR "airway s" OR "airways") AND ("exchangable" OR "exchange" OR "exchangeabilities" OR "exchangeability" OR "exchangeable" OR "exchanged" OR "exchanger" OR "exchanger s" OR "exchangers" OR "exchanges" OR "exchanging") AND ("catheter s" OR "catheters" OR "catheters" OR "catheter")) OR ("AEC") | 43,522 |
